# 

**Published:** 1846-04-01

**Authors:** 


*To our Readers.*—We have devoted the greater part of this number to subjects connected with medical reform, to the exclusion of other matter which we had accumulated for the department of Medical Intelligence, &c. We trust that the interest which our readers feel in these subjects renders an apology for this course uncalled for. After the present number we shall only devote to them the relative share of attention to which they are entitled. As we propose to issue on the first of next month simultaneously the last number of the present volume, and the first number of the second volume enlarged, we shall have space to make amends for the dearth of general medical intelligence in our present number. Publishers who have favored us with new publications will find them noticed at that time.

☞ The proceedings of the Buffalo City Association, and of the Erie Co. Medical Society with relation to the National Convention and the appointment of delegates, for want of space in the body of the work are placed on the cover, next page.

*We beg leave to call the attention of our readers to our circular, and particularly to the clause offering advantages to subscribers who remit before the first of May.*

☞ The Buffalo Medical Association will hold its stated monthly meeting on Tuesday evening, (7th inst.) at the office of Dr. Congar.

